# Small RNAs in Cnidaria: A review

**DOI:** 10.1111/eva.13445

**Published:** 2022-07-19

**Authors:** Yiqian Li, Jerome H. L. Hui

**Affiliations:** ^1^ Simon F.S. Li Marine Science Laboratory, State Key Laboratory of Agrobiotechnology, School of Life Sciences The Chinese University of Hong Kong Hong Kong City Hong Kong

**Keywords:** cnidarians, evolution, small RNAs

## Abstract

As fundamental components of RNA silencing, small RNA (sRNA) molecules ranging from 20 to 32 nucleotides in length have been found as potent regulators of gene expression and genome stability in many biological processes of eukaryotes. Three major small RNAs are active in animals, including the microRNA (miRNA), short interfering RNA (siRNA), and PIWI‐interacting RNA (piRNA). Cnidarians, the sister group to bilaterians, are at a critical phylogenetic node to better model eukaryotic small RNA pathway evolution. To date, most of our understanding of sRNA regulation and its potential contribution to evolution has been limited to a few triploblastic bilaterian and plant models. The diploblastic nonbilaterians, including the cnidarians, are understudied in this regard. Therefore, this review will present the current‐known small RNA information in cnidarians to enhance our understanding of the development of the small RNA pathways in early branch animals.

## 1 INTRODUCTION

Small RNA (sRNA) molecules are 20–32 nucleotides small noncoding RNA molecules, which have been found as potent regulators of gene expression and genome stability in many biological processes in eukaryotes (Ghildiyal & Zamore, 2009; Ishizu et al., 2012). They are classified into three major types: (1) microRNAs (miRNAs); (2) small interfering RNA (siRNAs); and (3) PIWI‐interacting RNAs (piRNAs), defined by their size and their association with Argonaute (AGO) family proteins (Ha & Kim, 2014). AGO proteins are the core proteins of small RNA pathways, which are further subdivided into the AGO and PIWI subfamilies. AGO proteins bind sRNA to form RNA‐induced silencing complexes (RISCs), which cause translational inhibition or cleavage of the complementary RNA targets (Swarts et al., 2014). The eukaryotes are likely to use the small RNA pathway mechanism to protect against exogenous viruses and transposons (Shabalina & Koonin, 2008). There is a wide range of functional and mechanistic diversities in eukaryotic small RNA pathways. However, detailed mechanistic studies have to date mainly been limited to a small range of bilaterian and plant models, and it is unclear how the existing small RNA pathways in extant metazoans evolved. Therefore, data from a broader range of organisms are needed to better understand the evolution of eukaryotic small RNA pathways.

Cnidaria is a class of invertebrates that includes a wide variety of morphologically and ecologically diverse animals, including sea anemones, corals, hydroids, and jellyfish. They have approximately 9000 member species which live in aquatic environments, mostly in the oceans (Technau & Steele, 2011). Cnidaria falls in an early branch of the metazoan lineage and is the sister group to bilateria. These two groups diverged ~600 million years ago (MYA) (Erwin et al., 2011). Cnidarians have recently emerged as the tractable genetic and molecular models for interfering animal evolution. The genomes and transcriptomes of many cnidarian species have been sequenced (Chapman et al., 2010; Gold et al., 2019; Khalturin et al., 2019; Kim et al., 2019; Leclère et al., 2019; Nong et al., 2020; Putnam et al., 2007; Shinzato et al., 2011), and some key methods such as mRNA injection, RNA interference, morpholinos, transgenics, in situ hybridization, live imaging, and gene editing have been utilized efficiently in cnidarians (Cleves et al., 2018; He et al., 2018; Technau & Steele, 2011). The bilaterians comprise >99% of extant described animal species, and to understand how bilaterian small RNA machineries evolved, comparisons to their outgroups could shed light. According to the phylogenomic studies, the closest sister group to the bilateria is generally considered as Cnidaria (Dunn et al., 2008) or a clade of Cnidaria plus Placozoa (Laumer et al., 2018). Thus, cnidarians provide an important outgroup to investigate aspects of the evolution of bilaterians small RNA function.

## 2 miRNAs

### 2.1 miRNA pathway in cnidarians

miRNAs are found in most eukaryotes, although they are not ubiquitous (De Jong et al., 2009; Gaiti et al., 2017; Moran et al., 2017; Moroz et al., 2014). They are 20‐ to 24‐nt long and are an abundant class of small RNAs involved in regulating numerous developmental and cellular processes in plants and animals through binding with complementary mRNA targets and recruiting effector proteins, which results in translational repression or target cleavage (Ameres & Zamore, 2013; Jonas & Izaurralde, 2015). The microRNA information from various evolutionary models has emerged as a potential source for resolving evolutionary relationships. Two evolutionary scenarios have been postulated based on the existing available data: (1) miRNAs developed convergently in animals and plants and have evolved at least nine times independently during eukaryotic evolution (Dinoflagellate, brown algae, green algae, land plants, excavates, molds, sponges, cnidarians, and bilaterians); or (2) miRNAs arose from a common ancestor of plants and animals and were subsequently lost in several lineages during evolution (Axtell, 2013; Moran et al., 2017; Tarver et al., 2012). Investigating the similarities and differences of miRNA pathways in a wide range of eukaryotes has been one effective method to resolve these problems. However, most previous research has been restricted to the bilaterian clade of metazoans and land plants which exhibits remarkable variations in small RNA pathways. For example, there is generally a lack of sequence similarity between the microRNAs of bilaterians and plants, and the loss of miRNAs families is common in various taxon of eukaryotes (Chinnappa et al., 2022; Fromm et al., 2013; Thomson et al., 2014). Among the nonbilaterians, only a few miRNAs have been identified in the sponge (Grimson et al., 2008; Liew et al., 2016; Robinson et al., 2013), and ctenophores do not have microprocessor complex and miRNAs (Maxwell et al., 2012; Moroz et al., 2014). The loss of microprocessor components is also observed in nonmetazoans such as fungi and Amoebozoa (Avesson et al., 2012; Bråte et al., 2018). These findings lead to a general perception that miRNA could have originated independently in different lineages. However, recent research in cnidarians suggests that the miRNA pathway might have originated from a common eukaryotic ancestor.

### 2.2 miRNA biogenesis

**FIGURE 1:**
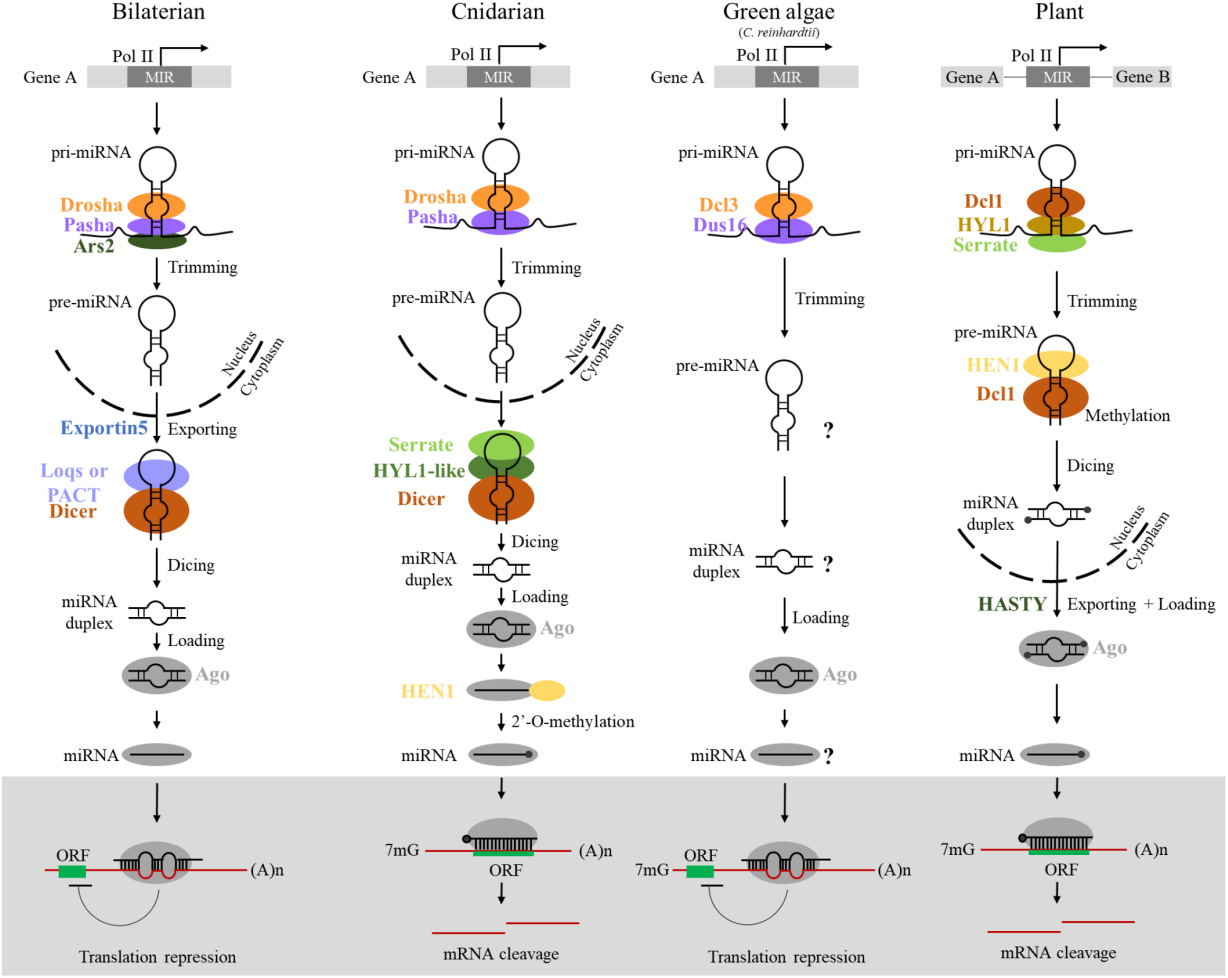
Cnidarian microRNA biogenesis pathways compared with bilaterians, green algae and plants. In cnidarians, pri‐miRNAs are proposed to be processed into pre‐miRNAs by the miRNA microprocessor complex composed of pasha and drosha. The loop structure is then removed by dicer, together with plant‐associated cofactors serrate and HYL1 to form the miRNA duplex. Hen1 methylates the 3′ end of the miRNA after miRNA loading and star strand ejection. 3′ ends methylation of miRNAs is common in both plants and cnidarians (Modepalli et al., 2018), whereas bilaterian miRNAs do not undergo this modification.

For miRNA biogenesis (Figure 1), miRNAs are derived from the stem‐loop precursor molecules (pri‐miRNAs) and are processed to pre‐miRNA by cleaving the hairpin structure in both animals and plants (Filipowicz, 2005; Moran et al., 2017). They are then loaded onto an AGO protein and targeted to the complementary mRNA. However, different partners are involved in this process. In animals, the pre‐miRNA is carried out by a specialized microprocessor complex made up of the RNAse III Drosha and the double‐stranded RNA‐binding protein Pasha (called DGCR8 in mammals). The pre‐miRNA is then exported to the cytoplasm where the second step of cleavage is performed by RNAse III Dicer, which is aided by a variety of double‐stranded RNA‐binding protein partners such as Loquacious (Loqs) in flies or TRBP and PACT in mammals (Fukunaga et al., 2012; Kim et al., 2009). In plants, the Dicer homologue Dicer‐like 1 (Dcl1) oversees both processing steps required for miRNA maturation supported by the double‐stranded RNA binding motif (DSRM)‐containing protein HYL1 (hyponastic leaves 1), Serrate and HEN1 in the nucleus. HEN1 is responsible for the methylation of the miRNA duplex (Yu et al., 2005). In both animals and plants, the miRNA duplex interacts with Argonaute proteins (AGOs) in the cytoplasm, forming the RNA‐induced silencing complex (RISC), to guide the cleavage and/or translation inhibition of the target genes.

Another distinction between animals and plants microRNAs is their genomic locations. In animals, ~30% of miRNA genes are found in introns of pre‐miRNAs; and on the contrary, plants generally have few intronic miRNA genes (Axtell et al., 2011). In the green algae *Chlamydomonas reinhardtii* and brown algae *Ectocarpus* miRNA, it is worth noting that more than half of its miRNA genes are encoded introns of protein‐coding genes, similar to the situations in animals (Figure 1) (Tarver et al., 2015; Valli et al., 2016).

In cnidarians, the miRNA encoding loci are comparable to those seen in mammals, with 30% of miRNA‐encoding genes embedded within introns of protein‐coding genes, and the majority of the rest located in intergenic regions (Fridrich et al., 2020) (Figure 1). However, cnidarians do not have homologues of the bilaterian Dicer partner proteins such as Loqs, TRBP, and PACT (Moran et al., 2013).

In plants, HYL1 is essential for regulating the microRNA biogenesis (Kurihara et al., 2006). Cnidarians encode the HYL1 homolog HYL1‐like a gene (HylLa), which is crucial for microRNA biogenesis in the sea anemone *Nematostella vectensis* (Moran et al., 2013; Tripathi et al., 2022 ). In 3′ ends of microRNAs of both plants and cnidarians, 2′‐O‐ methylation was carried out by HEN1 to improve miRNA stability, which is rarely found in other animals (Li et al., 2005; Modepalli et al., 2018; Zhao et al., 2012). Despite that HYL1‐like gene only acts with precursor miRNAs in the downstream step and not with primary miRNAs in cnidarians (Tripathi et al., 2022), given the fact that HYL1 homologues could also be identified in the other nonbilaterians such as sponges (Moran et al., 2013), these could well suggest that this protein was present in the microRNA system of the last common ancestor of animals and plants.

On the contrary, the Dicer‐like and plant‐like DSRM proteins in *Chlamydomonas* and various fungi are adequate for miRNA processing without the animal‐like accessary proteins such as Drosha and Pasha (Dias et al., 2017; Yamasaki et al., 2016). In cnidarians, homologues of both Drosha and Pasha could be identified, and Dicer is necessary for miRNA generation and normal development in the sea anemone *Nematostella* (Modepalli et al., 2018).

**FIGURE 2:**
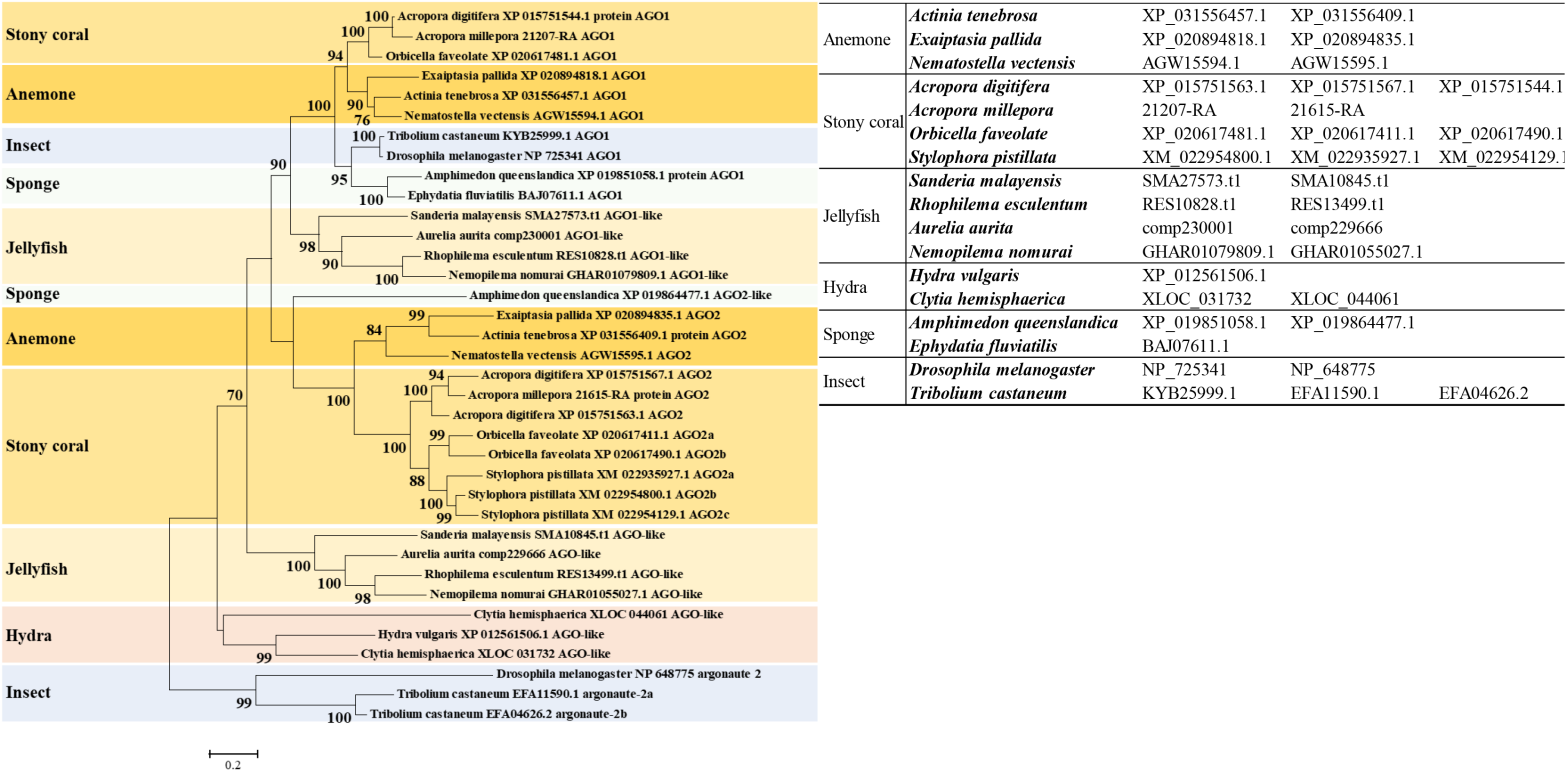
A phylogenetic relationship of metazoan Argonuate protein family. Multiple alignments were performed using MUSCLE and the rooted phylogenetic tree was constructed with the LG (G + I) model using the maximum likelihood method (with 1000 replicates). Bootstrap support values above 50% are indicated above branches.

In both animals and plants, the miRNA is eventually loaded into an AGO subfamily protein and regulates gene expression. AGO genes are found in a variety of copy numbers in eukaryotic genomes. These duplication events frequently result in sub‐ or neo‐functionalization (Lewis et al., 2016; Singh et al., 2015). Duplication events have also occurred in many cnidarian species with two or three AGO paralog proteins (Moran et al., 2013; Nong et al., 2020) (Figure 2). Both AGO paralogs of *Nematostella vectensis* are functional and bind to miRNA, but they have different miRNA sorting preferences (Fridrich et al., 2020). This independent duplication in cnidarians highlights the recurring trend of AGO duplication and subfunctionalization in eukaryotes, probably indicating its beneficial utility. These results suggest that cnidarian miRNA biogenesis mechanisms are comparable to both bilaterian and plant miRNA biogenesis mechanisms, which implies that the last common ancestor of eukaryotes might share a similar miRNA biogenesis machinery, which later significantly diverged in animals and plants.

### 2.3 miRNA regulation and conservation

Several studies have previously elucidated the molecular details of miRNA regulation in animals and plants. Plant miRNAs bind to their target RNAs through nearly perfect or perfect complementarity, which induces cleavage directly of their targets (Axtell et al., 2011). By contrast, bilaterian miRNAs mainly regulate the mRNAs via strong complementarity with the seed sequence, the nucleotides 2 to 8 at the 5′ end of the miRNA, which is required for target site recognition to the complementary sequences located in the 3′ untranslated regions of its target mRNAs. Base‐pairing of the seed region is adequate for binding and influencing the target mRNA. This overall low complementarity match inhibits the AGO protein from directly cleaving the RNA targets. On the contrary, AGO protein employs additional proteins that cause translational inhibition or transcript decay by deadenylation (Huntzinger & Izaurralde, 2011; Treiber et al., 2019). The distinct mechanism of plant and animal miRNA pathways supports the idea that miRNAs have evolved convergently. However, studies of *Nematostella* have convincingly established that miRNAs bind to their targets by nearly perfect complementarity, similar to siRNAs and plant miRNAs rather than bilaterian miRNAs (Moran et al., 2014; Figure 1).

The high complementarity base pairing between the miRNA and its target creates a strong response on target levels, but also imposes restrictions on possible targets. In fact, each plant and cnidarian miRNA has just a few targets compared to hundreds of possible targets regulated by a single animal miRNA (Ameres & Zamore, 2013; Axtell et al., 2011; Bartel, 2009; Rhoades et al., 2002). In the *Stylophora*, miR‐2022 has only two targets to be predicted (Liew et al., 2014). In mammals, by contrast, hundreds of transcripts are targeted by an average miRNA family (Friedman et al., 2009; Lewis et al., 2005). As a result, the loss of a miRNA would most likely affect the expression of multiple target genes, causing harm to the organism in bilaterians, while plants and cnidarians are more likely to withstand miRNA loss since a single miRNA only influences the expression levels of a limited number of target genes.

Nevertheless, the discovery of many miRNA‐directed cleavages in cnidarians does not exclude bilaterian‐like interactions between the miRNA seed and additional transcript targets. Cnidarian miRNAs may potentially target certain mRNAs via 12–15 nucleotides short match and, most likely, use translation inhibition as a mode of action (Baumgarten et al., 2018; Mauri et al., 2017; Praher et al., 2021). Under various circumstances or for different targets, the miRNA‐mediated silencing mechanism in cnidarians exploits both modes of action.

**FIGURE 3:**
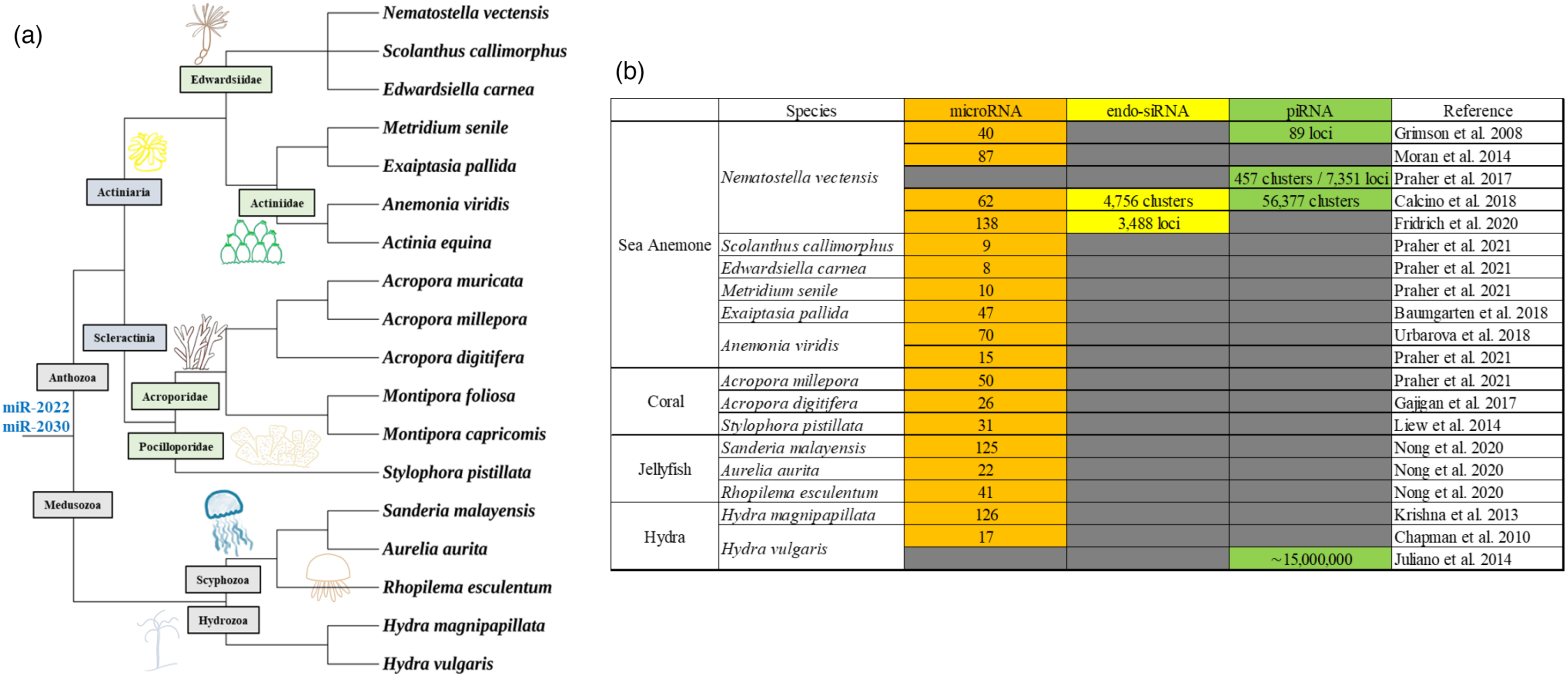
(a) the phylogenetic tree of cnidarian species with small RNA sequencing; (b) list of species of cnidaria small RNA research. The green, yellow and orange grids indicate that the small RNAs in question have already been the subject of study, while the gray grids indicate that no studies have yet been conducted.

Bilaterian miRNAs have a relatively high degree of conservation between species, and more than 30 conserved miRNAs have so far been identified (Dexheimer & Cochella, 2020; Fromm et al., 2022). By contrast, plant miRNAs evolved quickly, with a relatively high rate of gain and loss throughout evolution. In general, relatively few miRNAs are conserved in distantly related land plants and the majority of miRNAs are found to be lineage‐specific (Baldrich et al., 2018; Taylor et al., 2014; You et al., 2017). The situations of cnidarians are similar to that in plants. miRNAs have been sequenced in several cnidarian species including the sea anemone, coral, hydra and jellyfish (Figure 3) (Baumgarten et al., 2018; Calcino et al., 2018; Chapman et al., 2010; Fridrich et al., 2020; Gajigan & Conaco, 2017; Grimson et al., 2008; Juliano et al., 2014; Krishna et al., 2013; Liew et al., 2014; Lim et al., 2014; Modepalli et al., 2018; Modepalli & Moran, 2017; Moran et al., 2014; Nong et al., 2020; Praher et al., 2017, 2021; Teefy et al., 2020; Tripathi et al., 2022; Urbarova et al., 2018; Waldron et al., 2018; Zhu et al., 2021). The most well‐studied cnidarian model is *Nematostella vectensis*. The recent small RNA research has increased the number of its miRNAs from 40 to 138 (Fridrich et al., 2020; Grimson et al., 2008; Moran et al., 2014). miR‐100 is the only miRNA shared between bilaterians and cnidarians (Moran et al., 2014). Sequencing of small RNAs of sea anemone, hydroid and jellyfishes revealed only two conserved miRNAs (miR‐2022 and miR‐2030) in all investigated cnidarians (Krishna et al., 2013; Nong et al., 2020; Praher et al., 2021). These results also suggested a high turnover rate of miRNAs in the Cnidaria as compared to any other metazoans. It is worth noting that the sequence of *Nematostella* miR‐9422 is highly similar to that of plant miR‐156, a miRNA conserved from mosses to higher plants (Arazi et al., 2005; Moran et al., 2014). The lack of miRNA sequence conservation in cnidarians is also likely to be related to the ways in which miRNA regulates gene expression. However, the mechanisms by which miRNAs act to regulate gene expression in cnidarians remain largely unknown. It will be of great interest to investigate the roles individual miRNAs play in cnidarian developmental processes. For example, in a hydra head regeneration study, the expression profiles of several miRNAs have been shown to differ during the regeneration process, but no functional tests have yet been conducted to assess the efficacy of miRNA‐directed gene regulation in this species (Krishna et al., 2013).

## 3 OTHER SMALL RNAs

### 3.1 siRNA pathway in cnidarians

**FIGURE 4:**
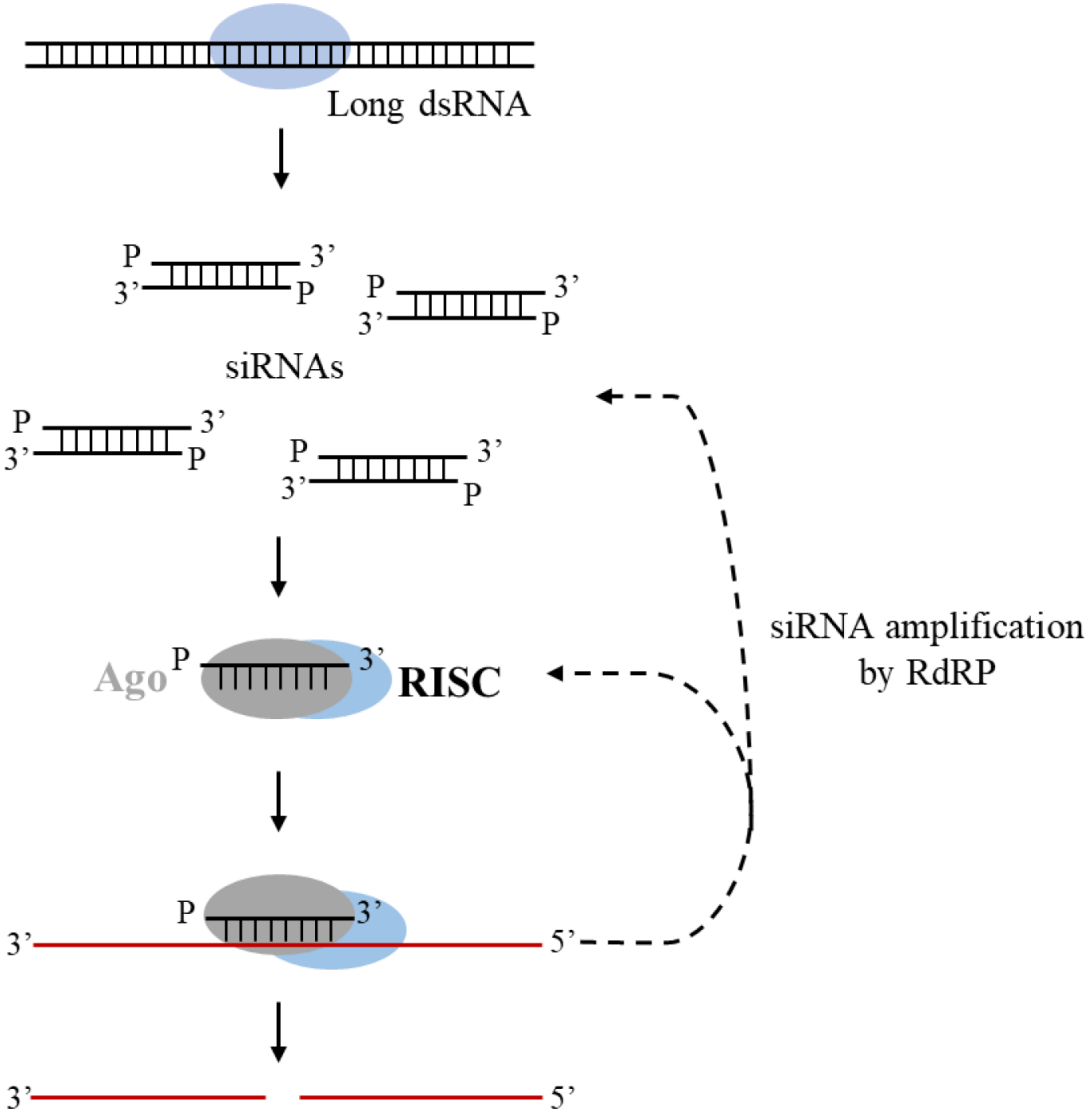
siRNA biogenesis. The ribonuclease III dicer dices dsRNA into short interfering RNAs (siRNA), which is often followed by signal amplification by RNA‐dependent RNA polymerases (RdRPs). When siRNAs are generated, they are bound by RISC, a multiprotein component complex (RNA‐induced silencing complex) and final silencing of foreign or invasive nucleic acids by Argonaute proteins.

Although there has been some study of the unresolved problems relating to miRNA evolution in cnidarians, less attention has been paid to the presence and roles of siRNA and piRNA systems in cnidarians. The siRNAs pathway is a primitive eukaryotic trait that most likely existed in the last common metazoan ancestor (Cerutti & Casas‐Mollano, 2006). siRNAs act as genome defenders in reaction to foreign or invasive nucleic acids such as viruses, transposons, and transgenes (Carthew & Sontheimer, 2009). The siRNAs share the overlapping biogenesis pathway and components with miRNAs, including Dicer and AGO, which are conserved in cnidarians. For siRNA biogenesis, mature siRNAs are processed by Dicers from longer double‐stranded RNA precursors, with numerous siRNA duplexes being created from both strands. The more stable single‐stranded siRNAs are then bound by multiprotein effector assemblies referred to as RISC, the RNA‐induced silencing complex. Finally, the siRNAs guide and align the RISC on the target mRNAs, and the mRNA is silenced by the catalytic RISC protein, which belongs to the argonaute family. The interaction between primary RISC and target mRNA causes RdRPs (RNA‐dependent RNA polymerases) to be recruited and secondary siRNAs to be synthesized from scratch utilizing the target mRNA as a template (Carthew & Sontheimer, 2009; Zhang & Ruvkun, 2012) (Figure 4).

In cnidarians, siRNA situations remain poorly characterized, and have only been reported by two species, *Hydra* and *Nematostella* (Fridrich et al., 2020; Krishna et al., 2013; Moran et al., 2014). As with other animals, the genes essential for siRNA biosynthesis such as dicers, argonautes and RdRPs could be identified in their genomes (Krishna et al., 2013; Zong et al., 2009). The most fruitful studies of the siRNA pathway have been conducted in *Nematostella*. siRNAs in *Nematostella* are preferentially enriched in NveAGO2, not NveAGO1, throughout development. The authors of these studies suggested that inverted duplication of the precursors results in highly complementary siRNA loading into NveAGO2. When mismatches accumulate and induce precursor mutation, the imperfectly matched precursors are processed as miRNAs and loaded into NveAGO1 (Fridrich et al., 2020). Similar to plants and bilaterians, where the main function of the siRNA pathway is to repress transposons and viruses, a previous study of RNAi in the early branch of metazoan animals including sponges, ctenophores, and cnidarians, demonstrated that both endo‐siRNA and piRNA clusters in these animals tend to map to transposons, suggesting that the function of siRNA in cnidarian is to silence TEs (Calcino et al., 2018). TEs are targets of the DNA methylation system in cnidarians, together with the fact that knocking down hywi gene in epithelial stem cells causes upregulation of TEs in *Hydra*, genome defense of TE has been suggested to relate to the longevity of cnidarians (Teefy et al., 2020; Ying et al., 2022).

### 3.2 PIWI‐piRNA pathway in Cnidarians

PIWI‐interacting RNAs (piRNAs) are a type of single‐stranded, 21–32 nucleotide long noncoding RNA that inhibit the mobilization of transposable elements which play essential roles in germ cell specification, embryonic patterning, neuronal activity and stem cell biology. (Rojas‐Ríos & Simonelig, 2018; Siomi et al., 2011). Unlike miRNAs and siRNAs, which are found in a wide range of eukaryotes, piRNAs are unique in metazoans (Grimson et al., 2008). Like the AGO protein for miRNA and siRNA pathway, the center of the piRNA pathway is another argonaute protein subfamily, P‐element–induced wimpy testis (PIWI) proteins. PIWI was first reported in drosophila and performed a pivotal role in maintaining the self‐renewal of germline stem cells (Lin & Spradling, 1997). It was later observed that PIWI proteins bind to a family of associated RNAs with lengths ranging from 24 to 33 nucleotides, known as Piwi‐interacting RNAs (Aravin et al., 2006). Many piRNA research has concentrated on drosophila and mice, which mainly express piRNAs in germline cells with a protective function for the germline genome against the transposable element (TE) invasion and mobilization (Siomi et al., 2011).TEs are mobile genetic elements found in all genomes and, when allowed to move, can cause genomic instability (Wessler, 2006). piRNAs bound to PIWI proteins are complementary base paired to targeted mRNAs, and target mRNAs are then cleaved by the slicing activity of the PIWI protein. This mechanism is similar to the other RNA‐based silencing events.

## 4 SOMATIC EXPRESSION

Although piRNAs repressing TE mobilization in the germline seems to be universal among metazoans, piRNAs are also found in a variety of somatic cells in various tissues and organisms (Ross et al., 2014). For example, the piRNA pathway has been expressed in drosophila somatic fat body cells, brain cells, and stem cells (Jones et al., 2016; Perrat et al., 2013; Sousa‐Victor et al., 2017). The somatic expression of piRNAs targeting TE mRNAs is also common among molluscs and arthropods (Jehn et al., 2018; Lewis et al., 2018). Although piRNA functions in bilaterian animals have been widely studied, few detailed studies in nonbilaterian animals such as cnidarians have been conducted. Studies conducted to date have considered the function of piRNAs in hydra, *Nematostella* sea anemone and the *Anemonia* sea anemone (Calcino et al., 2018; Juliano et al., 2014; Krishna et al., 2013; Lim et al., 2014; Moran et al., 2014; Praher et al., 2017; Teefy et al., 2020; Urbarova et al., 2018). Based on the data acquired so far, somatic PIWI‐mediated TE silencing appears to be common in cnidarian somatic tissues. This supports the hypothesis that somatic TE repression via the PIWI‐piRNA pathway represents an ancient metazoan trait that was later limited primarily to the germline of several metazoans.

## 5 CNIDARIANS SHARE CONSERVED piRNA BIOGENESIS MECHANISMS WITH BILATERIANS

The majority of cnidarians have two nonredundant PIWI genes (Praher et al., 2017). Unlike flies, mice and humans, which encode the PIWI genes in the nucleus with the functions of nuclear translocation and epigenetic inheritance, studies of hydras have indicated that the two PIWI proteins are located in the cytoplasm of hydra stem or progenitor cells (Juliano et al., 2014; Luteijn & Ketting, 2013). Two cnidarian species, *Nematostella* and *Hydra*, have been chosen as representative cnidarian models to study the piRNA‐PIWI pathway (Calcino et al., 2018; Juliano et al., 2014; Praher et al., 2017). piRNAs constituted the majority of the sequenced small RNAs in both species, as opposed to siRNAs and miRNAs.

**FIGURE 5:**
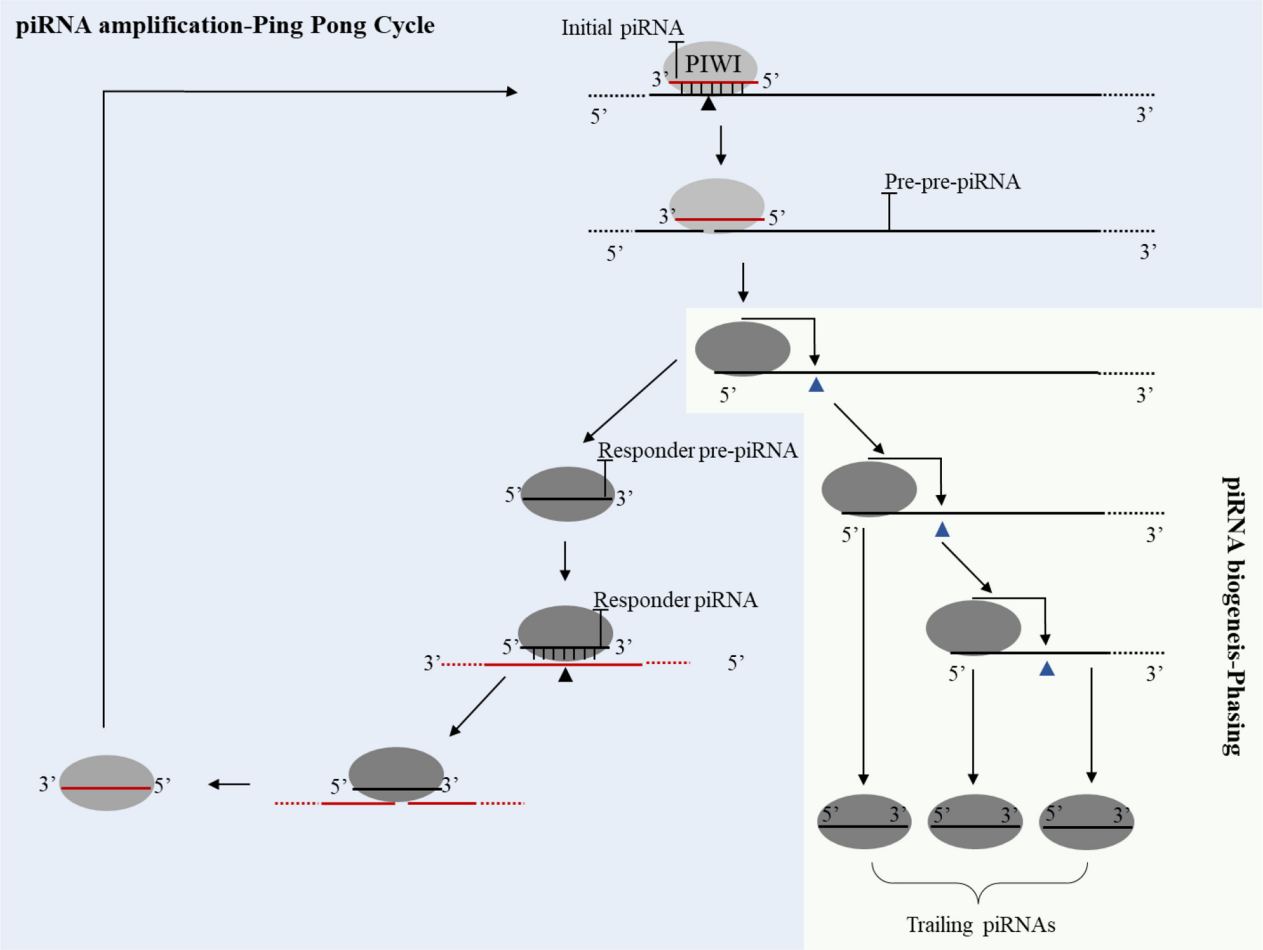
piRNA biogenesis and TE targeting. Initiator piRNAs guide PIWI‐catalyzed cleavage of a target piRNA precursor (black triangles). The sliced target is then loaded into another PIWI protein (dark ellipses) to produce trailing piRNAs by the piRNA‐independent endonuclease and pre‐piRNA 3′‐to‐5′ trimming exonuclease (dark blue triangles) during the phasing mechanism. Simultaneously, cleavage by initiator piRNAs also generates responder piRNAs which bind to target RNAs to guide cleavage and then in turn act as initiator piRNAs after loading to another PIWI to silence the piRNA precursors. This mechanism of piRNA amplification is called the ping‐pong cycle and is conserved in cnidarians.

Three types of piRNAs are represented in bilaterians: initiator piRNAs, responder piRNAs, and trailing piRNAs (Ozata et al., 2018). Cnidarians display conserved piRNA biogenesis mechanisms with other metazoans (Figure 5; Gainetdinov et al., 2018; Juliano et al., 2014). Cnidarian piRNAs, similar to most meiotic metazoans, are transcribed from uni‐strand piRNA loci or piRNA clusters. These clusters subsequently generate single‐stranded RNA molecules, which serve as the precursor for piRNAs and are complementary to TE sequences. Once produced, precursor piRNAs are loaded into a membraneless perinuclear organelle (known as nuage) for piRNA‐mediated transposon silencing (Ozata et al., 2018). piRNA processing in nuage is conserved among cnidarians, as evidenced by the presence of PIWI‐related nuage structures in both germline and somatic cells of *Hydra* and *Nematosttella* (Leclère et al., 2019; Lim et al., 2014; Praher et al., 2017).

To produce mature piRNAs, two routes work together: the ping‐pong pathway and the phased piRNA pathway (Figure 5). The ping‐pong route provides the initiator piRNAs that cleave the complementary targets to create responder piRNAs and launch the phased piRNA pathway's generation of trailing piRNAs. The uridine enrichment at the 5′ ends and the 2′‐O‐methylation modification at the 3′ end of the piRNA, which is mediated by the methyltransferase Hen1 in *Nematostella* and plays a crucial role in piRNA stability, are likewise conserved in cnidarians (Juliano et al., 2014; Modepalli et al., 2018; Ozata et al., 2018; Praher et al., 2017; Teefy et al., 2020). In sum, the mechanism of piRNA biogenesis is seen in several bilaterian species and a presentative cnidarian model Hydra, raising the possibility that bilaterians and cnidarians shared a similar piRNA biogenesis mechanism (Gainetdinov et al., 2018; Juliano et al., 2014).

## 6 FUNCTIONS FOR THE PIWI‐piRNA PATHWAY IN CNIDARIANS

Although the most well‐studied and conserved function of the PIWI‐piRNA pathway is transposable element repression, other functions have also been revealed. The PIWI‐piRNA pathway is necessary for *Hydra magnipapillata* head regeneration, although its mechanisms have yet to be elucidated (Krishna et al., 2013). In both *Nematostella* and *Hydra*, in addition to transposon expression, many piRNAs also map to protein‐coding genes such as the hydra non‐transposon targets involved in cell cycle regulation. However, no piRNA‐targeted transcripts are shared across *Nematostella* and *Hydra*, indicating that targeting protein‐coding genes has a high turnover rate and is unique to each species (Juliano et al., 2014; Lim et al., 2014; Praher et al., 2017).

## 7 CONCLUSIONS

An increasing number of small RNAs will undoubtedly be identified in many distinct and diverse species because of recent advances in deep sequencing technology and bioinformatics tools. These rapidly growing studies allow us to investigate and re‐evaluate the evolution stories of small RNAs. This review summarizes the current findings of the small RNA pathways in cnidarians, the sister group of bilaterians, and how it advances our knowledge of the evolution of the small RNA pathways in ancestral eukaryotes. The cnidarian miRNA pathway is comparable with the bilaterian and plant miRNA pathways, implying that the eukaryotic miRNA pathway evolved from a common ancestor. Somatic piRNA expression in cnidarians also supports the view that somatic PIWI function is an ancestral feature in metazoans. Despite advances in our knowledge of small RNA evolution and regulatory research in cnidarians, most studies are still descriptive. Further studies are expected to resolve several long‐standing conundrums, such as the functional interaction of small RNAs and their targets, the implications for the gene regulatory networks, the influence of specific cell types and their impact on the organism, and the functional importance of the small RNA pathways in a larger hierarchy of regulatory mechanisms. We expect new technologies that allow in‐depth analysis in animals to provide the means of answering these questions and yield a better knowledge of the role of small RNA in eukaryotes.

## CONFLICT OF INTEREST

The authors declare no conflict of interest.

## Data Availability

Not applicable.
